# Parliament and the Profession

**Published:** 1916-05-13

**Authors:** 


### PARLIAMENT AND THE PROFESSION.

### Tuberculous Soldiers.

The question of the treatment of tuberculous soldiers was again raised in the House of Commons. Mr. Glyn-Jones inquired how many men had been discharged from the Navy and Army during the past year because they were suffering from tuberculosis; and in what proportion of such cases had the Insurance Commission been able to provide immediate accommodation in sanatoria. Mr. Charles Roberts, who replied, did not supply the information asked for in the first part of the question. He said, however, that since the special arrangements for the provision of immediate residential treatment for discharged tuberculous sailors and soldiers came into force in April and August 1915 respectively, the several Commissioners had had approximately 2,450 cases reported to them by the War Office and Admiralty, as cases in which residential treatment was essential, and he was not aware of any such case in which accommodation had not immediately been made available.

### Tobacco and the Soldier's Heart.

The abuse of tobacco by soldiers and its effects were strikingly dealt with in an article which appeared in The Hospital of February 5 (p. 408), and in view of the state of affairs described by the writer it is not surprising that the attention of the House of Commons has been drawn to the fact that the efficiency of soldiers may be reduced by over-indulgence of this habit. Mr. J. M. Henderson asked the Under-Secretary for War whether there was a very large number of men of the Expeditionary Force in hospital with "what is called trench heart, but which is really the result of excessive cigarette and tobacco smoking." In his reply Mr. Tennant said that a certain number of cases of disordered action of the heart were occurring—the so-called "soldiers' heart." This, he said, was being investigated in a hospital specially set aside for the purpose. "By some observers," added Mr. Tennant, "this condition is attributed to excessive cigarette and tobacco smoking, and this is being looked into by skilled experts."

### Pharmacists in the Army.

The attitude of the War Office in the matter of Army dispensing is not easy to understand. As Mr. Tyson Wilson pointed out in a question he asked the Under-Secretary for War, there are a number of qualified chemists in the Army whose services are not being used to the best advantage. Mr. Wilson suggested that in view of the demand for dispensers the War Office should take steps in the interests of the Army to utilise the skill and knowledge of these men. Mr. Tennant replied that all medical units at home and abroad are fully supplied with "dispensers," and that if any qualified chemists who are serving in combatant units made application to be transferred to the Royal Army Medical Corps, the case of each applicant would be considered as vacancies for employment arose. This statement is not quite so satisfactory as it sounds. Apparently Mr. Tennant missed the point, for no one doubted but that all medical units were fully supplied with "dispensers"; the question is, are these "dispensers" properly trained and qualified pharmacists, and if not, why not, in view of the fact that a supply of such persons is available?

### Venereal Diseases.

Replying to Mr. King, the Prime Minister stated that the Government had decided to adopt the recommendations of the Royal Commission on Venereal Diseases as to the diagnosis and treatment of these diseases, and that arrangements were being made accordingly. He could not at the moment promise legislation on the other recommendations of the Commissioners, some of which raised very controversial questions, but the whole matter was receiving the earnest attention of the President of the Local Government Board.

### Insanitary Dwellings and Disease.

In reply to a question addressed to the President of the Local Government Board with regard to certain dwellings in the Southwark district which are alleged to be insanitary, Mr. Hayes Fisher said it was the duty of every local authority to make a closing order in respect of any dwelling-house which was in a state so dangerous or injurious to health as to be unfit for human habitation.

### Fee for Notifying Diseases.

The clause in the Local Government (Emergency Provisions) Bill which contains provisions as to the notification of disease in the form in which it has been passed in both Houses provides that the notification fee shall be "one shilling and no more."

### Other Matters of Interest.

The Clothing of Men Discharged from Hospital.—In a question he asked Mr. Tennant, Mr. M. Barlow suggested that certain soldiers who had been discharged from hospital had not been supplied with the normal service allowance of clothing, but Mr. Tennant intimated that if that were so the intentions of the War Office had been misinterpreted.

Dental Treatment of British Troops in India.—Mr. Chamberlain is in communication with the Government of India and the War Office with reference to the suggestion that adequate provision is not made for the dental treatment of British troops in India. In the opinion of some there is no need to go so far away from home to find evidence that the dental service ought to be improved.

Auxiliary Sick-berth Staff.—Sir C. Kinloch-Cooke complained that the calling up of the St. John Ambulance Brigade had materially affected promotion in the ranks of the sick-berth staff, but the Parliamentary Secretary to the Admiralty denied this. So far as he knew, it was not correct that men of the St. John Ambulance Brigade were placed over men of the active service sick-berth staff possessing greater qualifications and experience.

British Prisoners in Bulgaria.—Information from various private sources has reached this country concerning the treatment to which British prisoners of war are subjected in Bulgaria. A report on the subject has recently been received from the United States Minister at Sofia, and Mr. Malcolm suggested that this report should be published in order to reassure and inform the British public. Lord Robert Cecil said the suggestion would not be overlooked.

